# Preclinical evaluation and first in human study of Al^18^F radiolabeled ODAP-urea-based PSMA targeting ligand for PET imaging of prostate cancer

**DOI:** 10.3389/fonc.2022.1030187

**Published:** 2022-10-20

**Authors:** Ya’nan Ren, Chen Liu, Teli Liu, Xiaojiang Duan, Qian Zhang, Jiayue Liu, Pei Wang, Qian Guo, Xing Yang, Peng Du, Hua Zhu, Zhi Yang

**Affiliations:** ^1^ Medical College, Guizhou University, Guiyang, Guizhou, China; ^2^ Key Laboratory of Carcinogenesis and Translational Research, Ministry of Education/Beijing, Key Laboratory for Research and Evaluation of Radiopharmaceuticals, National Medical Products Administration, Department of Nuclear Medicine, Peking University Cancer Hospital and Institute, Beijing, China; ^3^ Department of Nuclear Medicine, Peking University First Hospital, Beijing, China; ^4^ Key Laboratory of Carcinogenesis and Translational Research, Ministry of education/Beijing, Department of Urology, Peking University Cancer Hospital and Institute, Beijing, China

**Keywords:** PSMA, oxalyldiaminopropionic acid-urea (ODAP-Urea) ligand, prostate cancer, [18F]AlF2+, clinical translational

## Abstract

**Purpose:**

This study aimed to introduce a novel [^18^F]AlF-labeled ODAP-Urea-based Prostate-specific membrane antigen (PSMA) probe, named [^18^F]AlF-PSMA-137, which was derived from the successful modification of glutamate-like functional group. The preclinically physical and biological characteristics of the probe were analyzed. Polit clinical PET/CT translation was performed to analyze its feasibility in clinical diagnosis of prostate cancer.

**Methods:**

[^18^F]AlF-PSMA-137 was maturely labeled with the [^18^F]AlF^2+^ labeling technique. It was analyzed by radio-HPLC for radiochemical purity and stability analysis *in vitro* and *in vivo*. The PSMA specificity was investigated in PSMA-positive (LNCaP) and PSMA-negative (PC3) cells, and the binding affinity was evaluated in LNCaP cells. Micro-PET/CT imaging was performed in mice bearing LNCaP or PC3 tumors. Thirteen patients with newly diagnosed prostate cancer were included for [^18^F]AlF-PSMA-137 PET/CT imaging. Physiologic biodistribution and tumor burden were semi-quantitatively evaluated and the radiation dosimetry of [^18^F]AlF-PSMA-137 was estimated.

**Results:**

The radiochemical yield of [^18^F]AlF-PSMA-137 was 54.2 ± 10.7% (n = 16) with the radiochemical purity over 99% and the specific activity of 26.36 ± 7.33 GBq/μmol. The binding affinity to PSMA was 2.11 ± 0.63 nM. [^18^F]AlF-PSMA-137 showed high cell/tumor uptake which can be specifically blocked by PSMA inhibitor. According to the biodistribution in patients, [^18^F]AlF-PSMA-137 was mainly accumulated in kidneys, lacrimal glands, parotid glands, submandibular glands and liver which was similar to the extensive Glu-Ureas based probes. A total of 81 lesions were detected in PET/CT imaging and over 91% of lesions increased between 1 h p.i. (SUVmean: 10.98 ± 18.12) and 2 h p.i. (SUVmean: 14.25 ± 21.28) (*p* < 0.001). Additionally, the probe showed intensive accumulation in lesions which provided excellent imaging contrast with the high tumor-to-muscle ratio of 15.57 ± 27.21 at 1 h p.i. and 25.42 ± 36.60 at 2 h p.i. (*p* < 0.001), respectively. The effective dose of [^18^F]AlF-PSMA-137 was estimated as 0.0119 ± 0.0009 mSv/MBq.

**Conclusion:**

An ODAP-Urea-based PSMA probe [^18^F]AlF-PSMA-137 was successfully prepared with high specificity and binding affinity to PSMA. Micro-PET/CT imaging study demonstrated its feasibility for prostate cancer imaging. Pilot clinical study showed its potential for delay-imaging and prostate cancer detection.

## Introduction

**Figure 1:**
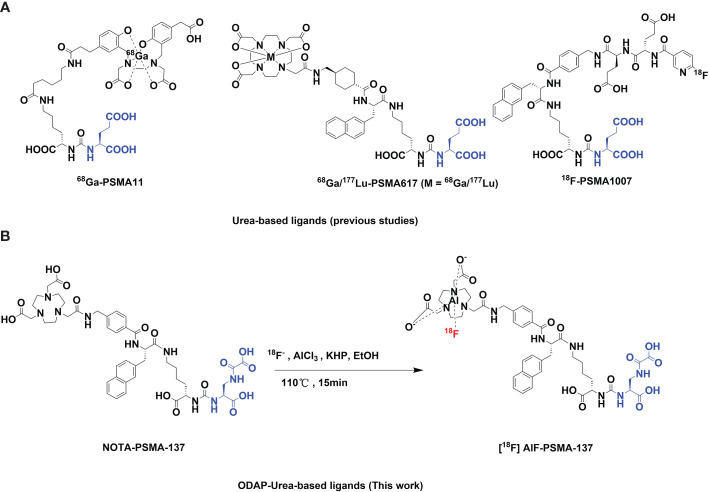
**(A)** Chemical structures of urea-based class PSMA inhibitors, ^68^Ga-PSMA-11, ^68^Ga/^177^Lu-PSMA-617 and ^18^F -PSMA-1007. **(B)** The radio-preparation process of a novel ODAP-Urea-based PSMA ligand in this work.

Prostate cancer (PCa) is the second most common malignant cancer among men, with a strong tendency of metastasis and heterogeneity, accounting for almost 1.4 million new cases and 375,000 associated deaths was estimated worldwide in 2020 (1). Prostate specific membrane antigen (PSMA) is an excellent target for specific imaging and targeted therapy for prostate cancer due to its overexpression in prostate cancer cells (2). Radiolabeled monoclonal antibodies targeting to PSMA such as J591 have been shown to be effective in PCa (3). However, the long half-life and poor tumor penetration of monoclonal antibodies is a significant limitation in imaging and therapy. Therefore, various small molecule inhibitors of PSMA have been reported over the past two decades, the most prevalent of which mimic the zinc-binding group of N-acetylaspartylglutamate (NAAG) in the catalytic pocket for imaging and therapy. PSMA targeted small molecule inhibitors can be mainly divided into three categories; urea-based, phosphorus-based, and thiol-based. Among them, urea-based inhibitors (**Figure 1A**) containing a Glu-Urea core motif that binds to PSMA protein with high efficiency and specificity currently dominate in clinical trials for specific imaging and radioligand therapy of PCa (4–13), such as ^68^Ga-PSMA-11 (the first PSMA agent for PET imaging approved by the U.S. Food and Drug Administration), ^68^Ga/^177^Lu/^225^Ac-PSMA-617, ^18^F-PSMA-1007, ^18^F-DCFPyL, Al^18^F-PSMA-BCH, ^99m^Tc-MIP-1404, ^99m^Tc-PSMA-I&S and ^18^F-rhPSMA-7.3 (14–18). Although the results are encouraging, the bladder residue due to the metabolic characteristics of the probes may affect the detection of intraprostatic lesions (19), making it necessary to develop new probes to optimize pharmacokinetic behavior.

In contrast to the extensive studies of PSMA ligands at the zinc binding site and S1 site, only a few studies reported the modification of the glutamate-like portion, and these reports were rarely successful due to the S1′ binding pocket of PSMA, which binds to the glutamate-like portion, has strict structural requirements to influence the binding affinity (20, 21). Of note, our collaborators have developed a novel type of oxalyldiaminopropionic acid-urea (ODAP-Urea) PSMA ligand, which represented a new modification strategy of the glutamate-like moiety binding to the S1’ sub-binding domain of PSMA. ^68^Ga-labelled ODAP-Urea based inhibitor showed similar or even higher PSMA binding potency than Glu-Ureas, [^68^Ga]Ga-PSMA-617, and has been successfully subjected to clinical study with lower bladder accumulation (22, 23). Therefore, there is great potential for the development of radionuclide labeled ODAP-Urea-based tracers for the diagnosis and therapy of PCa.

In comparison to ^68^Ga, ^18^F has an appropriate half-life (110 min vs. 68 min), as well as a higher positron emission (97% vs. 89%), contributing to higher sensitivity and spatial resolution (24). Furthermore, the cyclotron-produced ^18^F can be used for multi-person administration and emergency supply to adjacent departments, while the ^68^Ga is limited by generator production which can only meet 3-5 people at most (25). Due to the complexity and difficulty of the nucleophilic substitution reaction, the Al^18^F-labeling technique with NOTA chelator has been maturely developed with simplicity and controllability (26–28). Hence, we report a novel Al^18^F-labeled ODAP-Urea-based PSMA probe, [^18^F]AlF-PSMA-137, the preclinical biological behavior was studied and clinical translation was successfully carried out to provide valuable information for further diagnosis and therapy of PCa.

## Materials and methods

### Chemical reagents

The no-carrier-added Na^18^F was acquired from the Department of Nuclear Medicine, Peking University Cancer Hospital. The NOTA-PSMA-137 (MW = 964.5, **Figure S1**) precursor was synthesized using a solid phase platform as previously reported (22, 23). Aluminum chloride was purchased from Alfa Aesar Chemicals Co., Ltd (China). Potassium hydrogen phthalate (KHP) was purchased from Acros Organics (USA) and ethanol was purchased from Chemical Reagent (Sinopharm). Acetonitrile was purchased from Honeywell International Inc. (USA), and trifluoroacetic acid (TFA) was purchased from Shanghai Aladdin Biochemical Technology Co., Ltd (China). The reagents of cell culture including the medium, fetal bovine serum, PBS (phosphate buffer saline, pH 7.4, 0.01 M), trypsin, GlutaMAX, and penicillin–streptomycin solution were purchased from Biological Industries. ZJ-43 ((S)-2-(3-((S)-1-carboxy-3-methylbutyl)ureido), pentanedioic acid) was purchased from Tocris Bioscience.

### Radiochemistry and quality control

The radiolabeling process of [^18^F]AlF-PSMA-137 was based on a previously reported method with some modifications (29).^18^F- was captured on a pretreated (10 mL of 0.5 M NaHCO_3_ and 10 mL of water) anion-exchange cartridge QMA cartridge (Waters Corporation, USA) and eluted with 0.4 mL of saline. ^18^F- in saline (0.1 mL, 1.85–4.44 GBq), KHP (15 μL, 0.5 M, pH 4.0), AlCl_3_ (35 μL, 2 mM) in KHP (0.05 M), 100 μL anhydrous ethanol and 20 μL of NOTA-PSMA-137 (5 mg/mL) were mixed and then reacted at 110 °C for 15 min. After reaction, the solution was diluted with 3 mL of H_2_O and purified with a Sep-Pak C18-Light cartridge (Waters Corporation, USA), which was pretreated with 10 ml of ethanol and H_2_O. The final product was obtained by eluting the cartridge with 0.6 mL of 80% ethanol, diluting it with 5 mL of saline and passing through a 0.2 μm sterile filter membrane (Pall Corporation, USA) for further study.

Quality control of the final product was performed. The appearance was evaluated by visual inspection. Specific activity and ethanol content were roughly calculated. The pH value was determined by special indicator paper (Newstar, Hangzhou Shisan Science Co., Ltd, China). Radiochemical purity was determined by radio-high-performance liquid chromatography (radio-HPLC) (1200; Agilent, USA) with mobile phases of H_2_O (A) and acetonitrile (B) mixed with 0.1% trifluoracetic acid. Radio-HPLC was performed on a C18 cartridge (ZORBAX 300SB-C18, 4.6 mm × 250 mm, 5 μm; Agilent, Palo Alto, California, CA, USA) using a linear A–B gradient (0-10 min, 5%-60% of B, 10-15 min, 60%-40% of B) with a flow of 1 mL/min and 280 nm ultraviolet light.

### Partition coefficient and stability *in vivo* and *in vitro*

10 μL of [^18^F]AlF-PSMA-137 (1-2 MBq), 490 μL of phosphate-buffered saline (0.01 M, pH 7.4) and 500 μL of octanol was added into a tube. The mixture was vortexed for 3 min and centrifuged (3000 rpm, 5 min). Then, 5 samples (50 μL) from each phase were quantified using a γ-counter (Wizard II, Perkin Elmer Inc., Germany). The experiment was repeated 3 times. The value was expressed as logD7.4 (mean ± SD).

Stability *in vivo* and *in vitro* was studied by analyzing the radiochemical purity with radio-HPLC. For the *in vitro* stability, [^18^F]AlF-PSMA-137 was incubated in saline and 5% human serum albumin (HSA) for 1 h, 2 h and 4 h at 37°C. For *in vivo* stability assay, 37 MBq of [^18^F]AlF-PSMA-137 was injected into normal BALB/c mice, and the blood and urine were collected and treated at 5 min, 30 min and 60 min after injection.

### Cell culture and tumor models

The human prostate cancer cell lines, LNCaP and PC3, were obtained from the Chinese Academy of Sciences Typical Culture Collection (Shanghai, China). All of these cells were cultured in RPMI 1640 medium mixing with 10% fetal bovine serum (FBS), 1% penicillin−streptomycin and 1% GlutaMax-I, and then incubated in a humidified incubator at 37 °C with 5% CO_2_.

LNCaP cells (1×10^8^ cells/mL) suspension were prepared and placed at 4°C for pre-cooling, and then mixed with equal volume of pre-cooled Matrigel (Coring Incorporated, USA). After mixing, ice bath reserved and the cell suspension (0.2 mL/mouse, 1×10^7^ cells) were injected into the front leg of male NOD-SCID mice (HFK Bioscience Co., Ltd., Beijing). PC3 cells (0.1 mL; 1×10^7^ cells/mL) were injected into the front leg of male BALB/c nude mice (Vital River Laboratory Animal Technology Co., Ltd., Beijing). After 30-40 days of careful rearing of mice, the tumors grown to 5–10 mm in diameter and then the mice were used for biodistribution and micro-PET/CT imaging study.

### Cell uptake and binding affinity

The cell uptake assay was performed in PSMA-positive (LNCaP) and PSMA-negative (PC3) cells. The LNCaP and PC-3 cells were seeded in 24-well plates (pretreated with polylysine) (1×10^5^ cells/well) for 24 h before the cell uptake experiment. 500 μL of fresh medium with 74 kBq of [^18^F]AlF-PSMA-137 was added into the well plate (n = 4). After incubating for 5 min, 30 min, 1 h and 2 h respectively, the medium was removed and the cells were washed twice with 1 mL of cold PBS. The cells were lysed with 1 M NaOH, then the NaOH solution was collected and measured the radioactivity by γ counter. For blocking assay, 1 μg/well of ZJ-43 (a PSMA inhibitor) were co-incubated. The results were expressed as percentage injected activity per 1*10^5^ cells (% IA/10^5^ cells, mean ± SD).

The dissociation constant of [^18^F]AlF-PSMA-137 was performed on LNCaP cells by adding different concentrations of [^18^F]AlF-PSMA-137 (0.37-1.85 kBq/mL) into the 48-well plate (10^5^ cells/well, n = 4). After incubation for 1 h, the medium was removed, the cells were washed twice with cold PBS and then lysed by 0.2 mL NaOH. The radioactivity of NaOH solution was measured and the dissociation constant was calculated using the one-site total model of GraphPad Prism 8.3.0.

### Pharmacokinetics and biodistribution in mice

[^18^F]AlF-PSMA-137 (0.2 mL, 3.7 MBq) was intravenously injected into normal BALB/c male mice (n = 5) *via* the tail vein. The blood was collected from the ophthalmic artery and then weighed and measured for radioactivity by γ-counter at different time points. The results were expressed as the percentage of injected dose per gram (%ID/g). A two-compartment model was used to evaluate the blood pharmacokinetic behavior of [^18^F]AlF-PSMA-137, and the corresponding equation C*t* = Ae^−αt^ + Be^−βt^ was obtained by fitting the percentage of injection dose per gram (ID%/g) to time. A and B are the relevant constants for the model, and α and β are the rate constants of the distributed and eliminated phases, respectively.

For *in vivo* biodistribution in normal BALB/c male mice (n = 3) and NOD-SCID mice bearing LNCaP tumors (n = 4), [^18^F]AlF-PSMA-137 (0.2 mL, 0.74 MBq) was injected *via* the tail vein. The mice were sacrificed by cervical dislocation at different time points. For blocking study, mice bearing LNCaP tumors were co-injected with 50 ug of ZJ-43. The organs of interest were collected, weighted and measured for radioactivity by a γ-counter. Five samples of 1% injected dose were taken out and measured for radioactivity as a standard. The results were shown by calculating the percentage of injected dose per gram (%ID/g). The agent dose for pharmacokinetics and bio-distribution experiments was determined according to Wang et al. (30).

### Micro-PET/CT imaging studies

Micro-PET/CT imaging was performed for 10 min, followed by a CT scan (SNPC-303, Super Nova PET/CT, PINGSENG, Shanghai, China). Male NOD-SCID mice bearing LNCaP tumor, and BALB/c nude bearing PC3 tumor were injected with [^18^F]AlF-PSMA-137 (6.66 MBq-7.77 MBq, 0.2 mL) *via* tail vein, respectively. In mice bearing LNCaP tumor, 50 μg of ZJ-43 was co-injected with [^18^F]AlF-PSMA-137 for blocking. The images were obtained at 60 min, 120 min or 240 min p.i.

Images were analyzed using the Avatar software. After reconstruction of CT-AC PET with 3D-OSME+PSF algorithm, regions of interest (ROIs) were roughly drawn manually for delineation of the kidneys, heart, liver and tumor. The mean standardized uptake value (SUVmean) of interest organs was collected.

### PET/CT imaging in humans and radiation dosimetry estimation

The [^18^F]AlF-PSMA-137 PET/CT imaging study was approved by the Ethics Committee of Beijing Cancer Hospital (Ethical Approval Number: 2020KT154), and registered on ClinicalTrials.gov (NCT04693169). Thirteen patients were included in this study and all signed an informed consent form. The patients were intravenously injected with [^18^F]AlF-PSMA-137 (217.59 ± 21.16 MBq, 1.85-3.7 MBq/Kg). All patients were received PET/CT scans on a Biograph mCT Flow scanner (Siemens Healthineers, Erlangen, Germany) at 1 h and 2 h post injection (p.i.). Among them, four patients were additionally underwent PET/CT scans at 5 min p.i. and 3 h p.i. for further estimation of radiation dosimetry. The images were interpreted by two experienced physician and the SUVmax or SUVmean of physiological organs and tumor lesions were collected. The lesion was eventually confirmed as prostate cancer by pathology, comprehensive imaging or follow-up. When patients had less than or equal to ten lesions, all lesions were subjected to semi-quantitative analysis, if patients had more than ten lesions, ten measurable lesions were randomly selected for semi-quantitative analysis.

Average activity concentration (Bq/mL) of organs at 5 min, 1 h p.i., 2 h p.i and 3 h p.i. in 4 PCa patients were obtained by Siemens workstation (syngo.via Client 4.1) and the results were further analyzed with OLINDA/EXM software (version 2.0; Hermes Medical Solutions AB) to estimate the radiation dosimetry of each organ as well as the effective dose.

### Statistical analysis

Cell uptake, ROI-based quantification micro-PET/CT and PET/CT imaging data were analyzed using two-way ANOVA, multiple t tests or paired *t* test. Statistical analysis was performed using GraphPad software (GraphPad prism 8.3.0). The *p* values less than 0.05 considered statistically significant.

## Result

### Radiolabeling and quality control

[^18^F]AlF-PSMA-137 was prepared as shown in **Figure 1B**. The radio-labelling yield of was 54.2 ± 10.7% (n > 15). The radiochemical purity was >99% and the specific activity was 26.36 ± 7.33 GBq/μmol. As shown in **Table 1**, [^18^F]AlF-PSMA-137 injection passed the requirements of quality control.

**Table 1:** The quality control results of [^18^F]AlF-PSMA-137.

Parameter	QC specification	[^18^F]AlF-PSMA-137
Appearance	clear, colorless	Pass
Specific activity	18.5-296 GBq/μmol	26.36 ± 7.33 GBq/μmol
pH	4.0-7.0	5.8-6.4
Radio-HPLC	>95%	>99%
Ethanol	<10%	<10%
Endotoxins	<15 EU/mL	Pass
Sterility	Sterile	Pass

### Partition coefficient and stability

The LogD_7.4_ value of [^18^F]AlF-PSMA-137 was -2.12 ± 0.02, indicating that [^18^F]AlF-PSMA-137 was highly hydrophilic.

The *in vitro* and *in vivo* stability of [^18^F]AlF-PSMA-137 were evaluated by radio-HPLC. The radiochemical purity was > 99% after incubation in saline or in 5% HSA at 37°C for 4 h (**Figure S2A**). In addition, the radiochemical purity of [^18^F]AlF-PSMA-137 was >99% in the blood and urine of BALB/c mice until 60 min p.i. (**Figure S2B**). These results indicated that [^18^F]AlF-PSMA-137 was stable both *in vivo* and *in vitro*, and could be used for further study.

### Cell uptake

**Figure 2:**
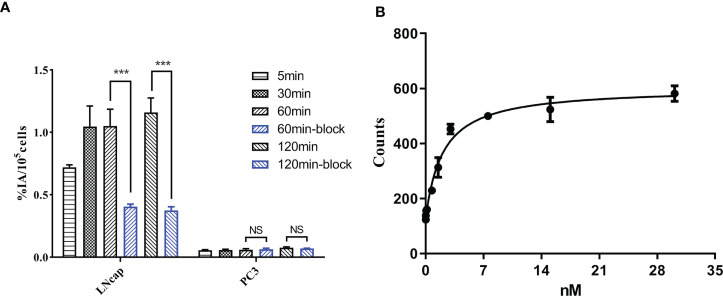
**(A)** The cell uptake of [^18^F]AlF-PSMA-137 in PSMA-positive LNCaP and PSMA-negative PC3 cells. **(B)** The dissociation constant of [^18^F]AlF-PSMA-137 in LNCaP cells was 2.11 ± 0.63 nM. Block = 1 μg of ZJ-43. ***P<0.001, NS, No significance.

As shown in **Figure 2A**, the uptake of [^18^F]AlF-PSMA-137 in LNCaP cells (1.16 ± 0.10%IA/10^5^ cells at 2 h) was obviously higher than that in PC3 cells (0.08 ± 0.01%IA/10^5^ cells at 2 h). Furthermore, when co-incubated with PSMA inhibitor, ZJ-43, the uptake in LNCaP cells (-67.70%, p<0.001) at 2 h was significantly decreased, while the uptake in PC3 cells (p>0.05) was not affected. The dissociation constant of [^18^F]AlF-PSMA-137 determined in LNCaP cells was 2.11 ± 0.63 nM (**Figure 2B**), which indicated [^18^F]AlF-PSMA-137 had high affinity to PSMA.

### Pharmacokinetics in blood and biodistribution

The pharmacokinetic formula of [^18^F]AlF-PSMA-137 in blood circulation was *C_t_* = 23.031e^-0.721t^ + 11.477e^-0.026t^, with a half-life of 0.961 min for distribution phase and 26.43 min for elimination phase (**Figure S3**).

**Figure 3:**
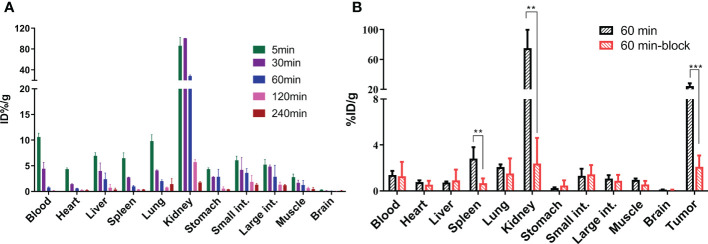
The organ biodistribution of [^18^F]AlF-PSMA-137 **(A)** in healthy BALB/c male mice (n = 3) at 5 min, 30 min, 60 min, 120 min and 240 min p.i. and **(B)** in mice bearing LNCaP tumor (n = 4) at 60 min p.i. int. = intestine. block = 50 μg of ZJ-43/mouse. **p < 0.01, ***p < 0.001.

As shown in **Figure 3A**, in normal BALB/c male mice, [^18^F]AlF-PSMA-137 showed rapid distribution along with blood (10.60 ± 0.74%ID/g) throughout the whole body with relatively high accumulation in all organs at 5 min p.i. and then decreased rapidly over time expect in the kidneys. At 30 min p.i., the tracer showed the highest uptake in kidneys (100.58 ± 0.15% ID/g) and relatively low uptake in most organs, such as heart (1.41 ± 0.11% ID/g), brain (0.16 ± 0.01% ID/g) and muscle (1.69 ± 0.51% ID/g) and then quickly cleared through the kidneys over time (1.74 ± 0.19% ID/g at 4 h p.i.). In mice bearing LNCaP tumors, [^18^F]AlF-PSMA-137 showed high accumulation in the kidneys (75.19 ± 20.04% ID/g) and tumor (24.29 ± 3.06% ID/g) at 1 h p.i., which could be specifically blocked by ZJ-43 (block value: kidneys: 2.39 ± 1.92% ID/g, *p* = 0.0017, tumor: 2.09 ± 0.81% ID/g, *p* = 0.0006) (**Figure 3B** and **Table S1**). The high tumor uptake of [^18^F]AlF-PSMA-137 also contributed to a high tumor-to-organ ratio, such as the tumor to kidney ratio was 0.37 ± 0.07 and tumor to muscle ratio was 23.31± 2.15 (**Table S1)**.

### Micro-PET/CT imaging studies

**Figure 4:**
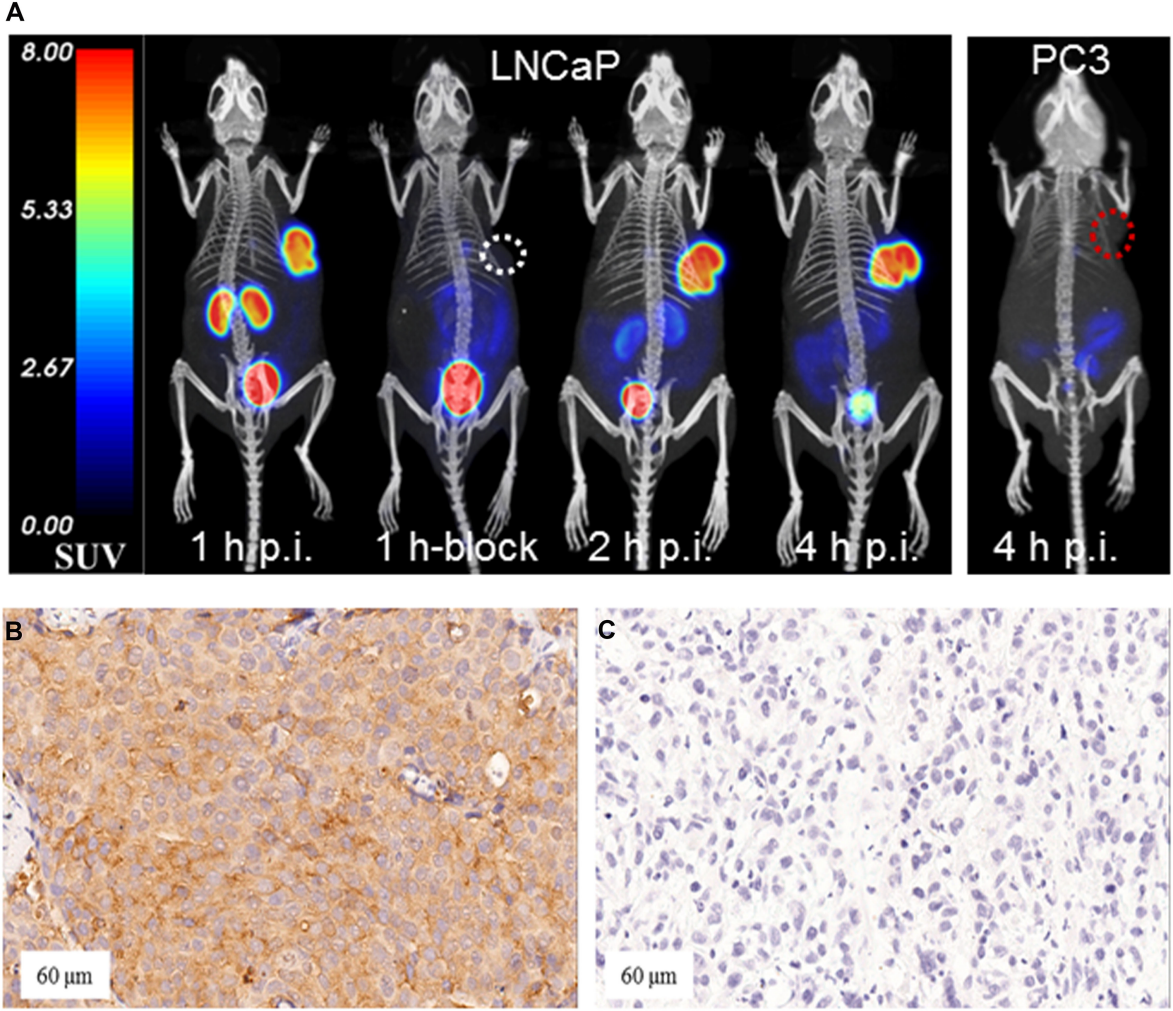
**(A)** Micro- PET/CT images in mice bearing LNCaP and PC3 tumor xenografts. White and red circles indicated LNCaP and PC-3 tumor. Immunohistochemical staining of **(B)** LNCaP xenografts (PSMA–positive) and **(C)** PC3 xenografts (PSMA–negative) (20×amplifcation. Block: co-injected with 50 μg of ZJ-43.

[^18^F]AlF-PSMA-137 mainly accumulated in the kidneys and PSMA (+) LNCaP tumor, followed by the bladder, intestinal tract and gall bladder, whereas the PSMA (-) PC3 tumor was almost invisible (**Figure 4A**). Radioactive accumulation in LNCaP tumor increased between 1 h p.i. (SUVmean: 7.79 ± 0.32) and 2 h p.i. (8.64 ± 0.08), and then slightly decreased at 4 h p.i. (8.57 ± 0.05), which was significantly higher than that of PC3 tumor (SUVmean: 0.10 ± 0.01 at 4 h p.i., *p* < 0.001) (**Figure S4A**). The uptake in kidneys decreased from 1 h p.i. (SUVmean: 9.74 ± 0.08) to 4 h p.i. (SUVmean: 1.32 ± 0.04). Besides, the uptake in LNCaP tumor (SUVmean: 0.35 ± 0.02, *p <*0.001) and kidneys (SUVmean: 1.34 ± 0.08, *p* < 0.001) could be significantly blocked when co-injecting with 50 μg of ZJ-43 at 1 h p.i. In addition, due to the intensive accumulation of [^18^F]AlF-PSMA-137 in tumor and the fast clearance from the normal organs, the images with high tumor-to-non-target organ ratios resulted to relatively good tissue contrast. Generally, the ratios of tumor-to-organ of [^18^F]AlF-PSMA-137 increased over time, and the excellent tumor-to-muscle ratio (323.88 ± 2.94) and tumor-to-kidney ratio (2.89 ± 0.09) were obtained at 2 h p.i. (**Figure S4B**). Furthermore, immunohistochemical staining results demonstrated that the high PSMA expression in LNCaP xenografts and low expression in PC3 xenografts (**Figures 4B–C**), which further verified the PSMA-targeting of [^18^F]AlF-PSMA-137.

### PET/CT imaging in prostate cancer patients

The clinical characteristics and PSMA PET/CT detected lesions of patients were shown in **Table 2**. [^18^F]AlF-PSMA-137 were tolerated well by all patients. No adverse events were reported.

**Table 2:** Characteristics of all patients investigated in this study.

Patient No.	Age (y)	Weight (Kg)	PSA (ng/mL)	Injected dose (MBq)	Gleason score	Primarytumor	LNMetastases	BoneMetastases	Soft-tissue
1	65	85	12.20	171.31	4+5	1	6	0	0
2	52	95	37.88	213.49	4+4	1	3	>10	0
3	58	65	111.7	139.49	5+5	1	>10	>10	0
4	67	87	48.01	263.07	4+4	3	3	0	0
5	63	75	86.32	226.81	3+4	3	9	0	0
6	69	70	41.24	209.79	4+4	2	0	2	>10
7	57	65	130.29	210.90	4+3	1	0	0	0
8	72	73	18	223.48	3+3	0	0	0	0
9	68	82	7.96	220.89	3+3	1	0	0	0
10	72	74	21.49	217.93	4+5	1	0	0	0
11	68	82	13.91	242.35	4+4	2	0	0	0
12	73	67	18	207.57	4+5	1	0	0	0
13	70	70	47.17	203.50	3+4	1	0	0	0

As shown in **Figure 4A** and **Table S2**, [^18^F]AlF-PSMA-137 showed intensive uptake in kidneys, lacrimal glands, parotid glands, submandibular glands, liver and bladder, with the SUVmean values of 10.67 ± 2.17, 4.31 ± 0.91, 8.83 ± 1.74, 9.31 ± 2.13, 8.59 ± 1.21 and 8.34 ± 5.50 at 1 h p.i. The uptake in kidneys (13.52 ± 2.93, *p* = 0.0004), lacrimal glands (4.97 ± 0.93, *p* = 0.0011), parotid gland (11.20 ± 2.60, *p* = 0.0003), submandibular gland (10.95 ± 2.82, *p* = 0.0039) and liver (9.58 ± 1.56, *p* = 0.0011) were significantly increased at 2 h p.i., while that in the bladder was significantly decreased (5.02 ± 2.06, *p* = 0.02). Other organs such as brain, lung, muscle, etc., and showed relatively low uptake and did not change between 1 h p.i. and 2 h p.i.

**Figure 5:**
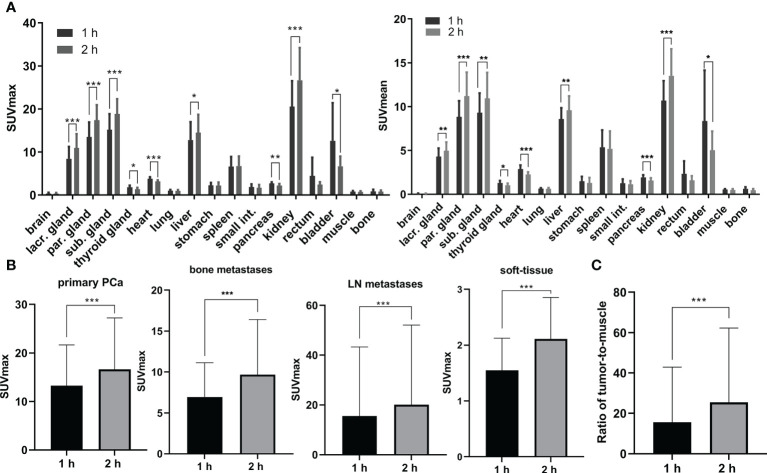
**(A)** The physiological biodistribution with average SUVmax and SUVmean in prostate cancer patients. **(B)** The average SUVmax in lesions including primary PCa, bone metastases, lymph node (LN) metastases and soft-tissue metastases at 1 h p.i. and 2 h p.i.. **(C)** The ratio of tumor-to-muscle in 12 PCa patients at 1 h p.i. and 2 h p.i.. lacr., lacrimal; par., parotid; sub., submandibular; int., intestine. *: p < 0.05, **: p < 0.01, ***: p < 0.00.

PSMA PET/CT imaging in 12 patients showed positive lesions in the prostate and 1 patient with Gleason score of 3 + 3 was negative. Totally, as shown in **Figure 4B** and **Figure S5**, 81 lesions including 18 primary PCa lesions, 22 bone metastases, 31 lymph node (LN) metastases and 10 soft-tissue metastases were observed. The average SUVmax values of primary PCa lesions, bone metastases, LN metastases and soft-tissue metastases were 13.25 ± 8.17 (range: 4.1-28.7), 6.94 ± 4.09 (range: 2.1-13.4), 15.57 ± 27.26 (range: 2.3-148.6) and 1.55 ± 0.55 (range: 0.9-2.5) at 1 h p.i. and then significantly increased at 2 h p.i. with average SUVmax values of 16.59 ± 10.32 (range: 4.3-35.3, *p* = 0.0002), 9.67 ± 6.58 (range: 2.5-16.5, *p* = 0.0004), 20.06 ± 31.49 (range: 3.1-167.5, *p* < 0.001) and 2.11 ± 0.71 (range: 1.3-3.4, *p* < 0.001), respectively. Among 18 primary lesions, the SUVmax of 44.44% lesions (n = 8) was significantly increased (> +30%) at 2 h p.i. and 55.56% lesions (n = 10) maintained (+30% ~ -30%) the same level as 1 h p.i. Among 63 metastatic lesions, the SUVmax values of 59.09% bone metastases lesions (n = 13), 58.06% LN metastases lesions (n = 18) and 70% soft-tissue metastases (n = 7) were significantly increased (>30%), while other lesions maintained (+30% ~ -30%) between 1 h p.i. and 2 h p.i. Accordingly, the low uptake in background (muscle: with average SUVmax values of 0.80 ± 0.15 at 1 h p.i. and 0.72 ± 0.20 at 2 h p.i.) and high accumulation in lesions contributed to the high ratio of tumor-to-muscle (15.57 ± 27.21 at 1 h p.i. and 25.42 ± 36.60 at 2 h p.i., *p* < 0.001) which provided a high image contrast (**Figure 5C**).

**Figure 6:**
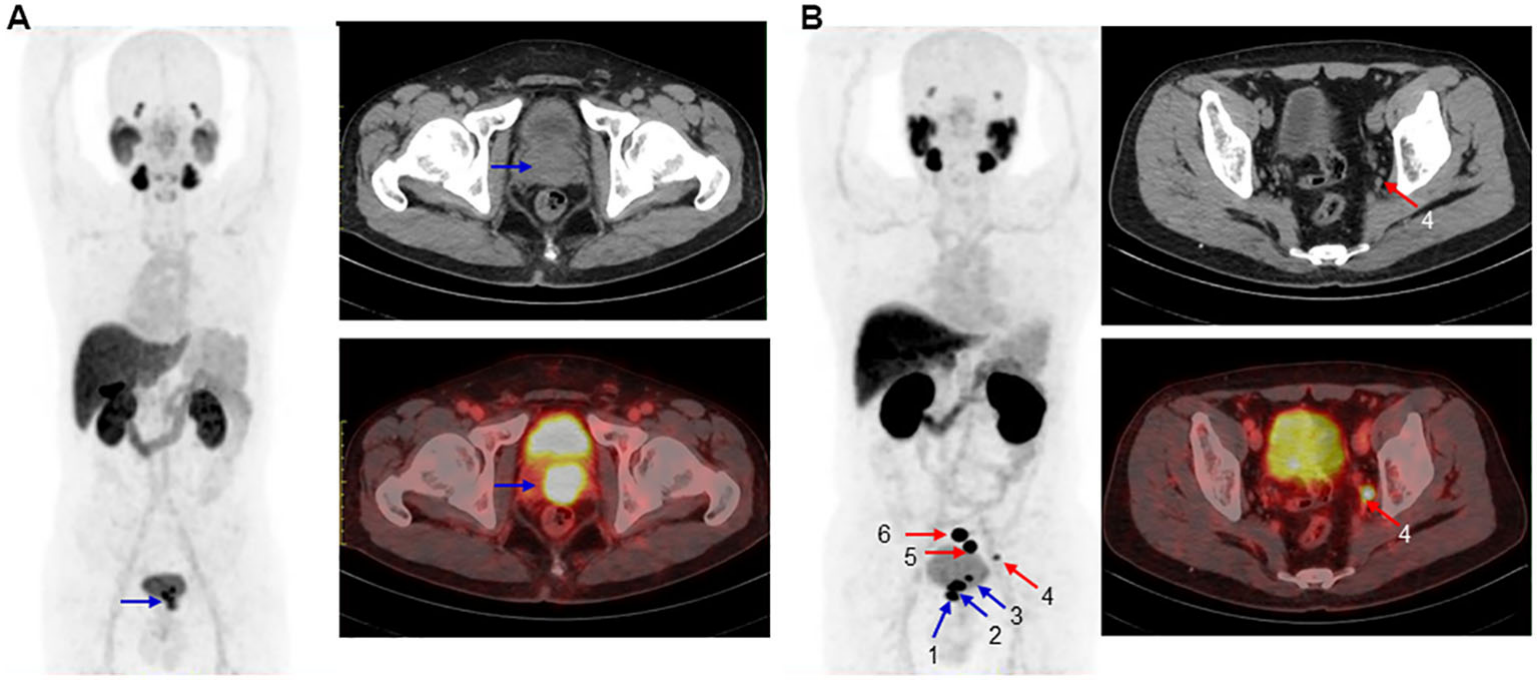
Maximum-intensity projections (left), CT image (top right) and PET/CT image (bottom right) of 2 patients at 1 h p.i.. **(A)**. In a 57 years man of No.7 with PSA of 130.29 ng/mL and Gleason score of 4+3=7. The blue arrow indicated primary PCa (SUVmax: 13.5). **(B)**. In a 67 years old patient No.4 with PSA of 48.01 ng/mL and Gleason score of 4+4=8. The blue and red arrows indicated primary PCa (primary 1, 2 and 3 with SUVmax of 28.7, 25.4 and 17) and multiple lymph node metastases (SUVmax rang: 10.4–149.4), respectively. CT and PET/CT imgaes showed the metastasis 4 with SUVmax of 10.4.

[^18^F]AlF-PSMA-137 PET/CT imaging could effectively detect the primary PCa and metastatic lesions at 1 h p.i. As shown in **Figure 5**, **6**, the primary tumor lesion in the left lobe base to apex showed intensive uptake with SUVmax of 13.5 (**Figure 6A**). On the MIP image of patient No.4, 3 primary tumor lesions (SUVmax range: 17-28.7) in the base to cusp right peripheral band showed intensive uptake and there were multiple lymph node metastases (SUVmax range: 10.4-149.4) in the mesenteric region, pararectal artery, and left obturator region (**Figure 6B**). In addition, [^18^F]AlF-PSMA-137 imaging clearly displayed the metastatic lesions at 2 h p.i. As shown in **Figure 6**, all lesions showed higher uptake and the blood pool significantly decreased at 2 h p.i.

### Estimation of radiation dosimetry in humans

Multi-phase imaging (**Figure S6**) was performed in 4 PCa patients in order to estimate internal radiation dosimetry. As shown in **Table 3** and **Table S3**, most organs presented low absorbed dose. The kidneys were the most irradiated organ with an absorbed dose of 1.13E-01 ± 2.37E-02 mGy/MBq (range: 9.06E-02–1.51E-01 mGy/MBq), followed by the salivary glands and spleen with absorbed doses of 5.68E-02 ± 9.39E-03 mGy/MBq (range: 4.49E-02–6.71E-02 mGy/MBq) and 4.83E-02 ± 9.81E-03 mGy/MBq (range: 3.32E-02–5.91E-02 mGy/MBq), respectively. The average effective dose was 0.0119 ± 0.0009 mSv/MBq (range: 0.0110–0.0133 mSv/MBq).

**Table 3:** Radiation dosimetry estimation of [^18^F]AlF-PSMA-137 in PCa patient (n = 4).

Target Organ	Absorbed Dose (mGy/MBq)		
	Mean		SD
Adrenals	2.47E-02		3.82E-03
Brain	2.52E-03		2.53E-04
Esophagus	9.38E-03		7.26E-04
Eyes	4.01E-03		6.16E-04
Gallbladder Wall	3.64E-02		6.74E-03
Left colon	8.80E-03		1.21E-03
Small Intestine	7.75E-03		1.11E-03
Stomach Wall	1.41E-02		3.17E-03
Right colon	9.48E-03		9.88E-04
Rectum	6.28E-03		1.12E-03
Heart Wall	2.10E-02		1.87E-03
Kidneys	1.13E-01		2.37E-02
Liver	4.40E-02		5.49E-03
Lungs	1.28E-02		1.03E-03
Pancreas	1.04E-02		1.08E-03
Prostate	6.75E-03		1.20E-03
Salivary Glands	5.68E-02		9.39E-03
Red Marrow	6.36E-03		6.51E-04
Osteogenic Cells	1.07E-02		1.20E-03
Spleen	4.83E-02		9.81E-03
Testes	4.50E-03		8.86E-04
Thymus	7.81E-03		5.85E-04
Thyroid	1.49E-02		2.05E-03
Urinary Bladder Wall	1.87E-02		5.84E-03
Total Body	6.91E-03		8.17E-04
Effective Dose (mSv/MBq)	1.19E-02		8.70E-04

## Discussion

With the advent of prostate specific membrane antigen (PSMA), many radioactive tracers targeting PSMA have been emerged, revolutionizing the diagnosis and treatment of prostate cancer. Most of them were ^68^Ga- and ^18^F-labeled Glu-Ureas based inhibitors, for instance, ^68^Ga-PSMA-11, ^68^Ga-PSMA-617 and ^18^F-PSMA-1007 (**Figure 1A**) with high detection efficiency have been reported (14, 31, 32). However, these probes were restricted to mimicking the zinc-binding group of N-acetylaspartylglutama. ^68^Ga-labeled ODAP-urea-based PSMA tracer that offset the modification threshold of PSMA ligands at the S1’ site binding to glutamate-like moiety have been reported (22, 23).

Here, in view of the excellent decay properties of ^18^F (T_1/2_, 109.77 min; β^+^, 96.7%) and the mature labeled strategy of [^18^F]AlF^2+^, we labeled the tracer with [^18^F]AlF^2+^. Both preclinical evaluation and clinical translation were performed to evaluate the potential of [^18^F]AlF-PSMA-137 for PCa imaging.

[^18^F]AlF-PSMA-137 was rapidly prepared with high radiochemical yield of 54.2 ± 10.7%, significantly higher than most of the previously reported [^18^F]AlF^2+^ labeled probes, such as [^18^F]AlF-PSMA-BCH (32.2% ± 4.5%) and [^18^F]AlF-NOTA-FAPI (33.8% ± 3.2%) (29, 30). It was reported that this may be due to the co-reaction with anhydrous ethanol (28). [^18^F]AlF-PSMA-137 was quality controlled and the solution met the criteria.

[^18^F]AlF-PSMA-137 showed obviously higher uptake in PSMA (+) LNCaP cells and tumor than that in PSMA (-) cells and tumor. The uptake in LNCaP cells and tumor was increased between 1 h and 2 h, and decreased by the addition of PSMA inhibitor, ZJ-43. Indicating [^18^F]AlF-PSMA-137 can specifically target to PSMA. The Kd value of 2.11 ± 0.63 nM measured with LNCaP cells demonstrated high affinity of [^18^F]AlF-PSMA-137. The value was slight higher than that of the urea-based inhibitors [^18^F]AlF-PSMA-11 (Kd value of 2.95 ± 0.87 nM in LNCaP cells) and [^18^F]AlF-PSMA-BCH (2.90 ± 0.83 nM in 22Rv1 cells) (8, 12).

**Figure 7:**
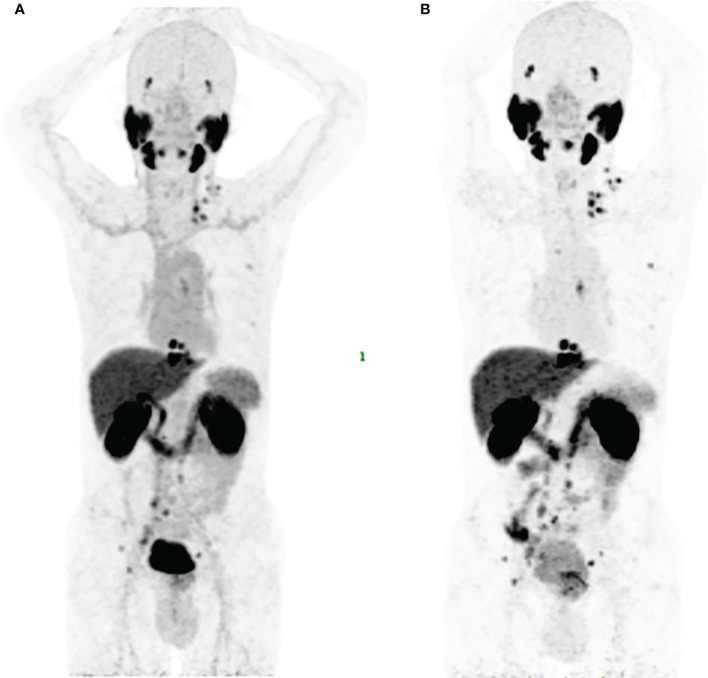
Maximum-intensity projections in a 58 years man of No.3 with PSA of 111.7 ng/mL and Gleason score of 5+5=10 at 1 h p.i. **(A)** and 2 h p.i. **(B)**.

As shown in **Figures 3**, **7**, [^18^F]AlF-PSMA-137 was mainly accumulated in kidneys and bladder, and the co-injection of ZJ-43 decreased the uptake of tracer in the kidneys indicating the high uptake of [^18^F]AlF-PSMA-137 in the kidneys was due to the expression of PSMA, as well as the metabolism of tracer from urinary tract.[30,31] [^18^F]AlF-PSMA-137 showed low uptake and fast clearance from non-target organs of mice, such as blood, heart, liver, lung and muscle which coincided with the short half-life (26.43 min) measured by the pharmacokinetic study. Bone was invisible in micro-PET/CT images, indicating that [^18^F]AlF-PSMA-137 was stable *in vivo* without dissociation of ^18^F from the tracer. This can be supported by the high *in vivo* stability of [^18^F]AlF-PSMA-137. The high PSMA (+) tumor-to-non-target organ ratios indicated that [^18^F]AlF-PSMA-137 was appropriate for tumor detecting. In the preclinical studies, no acute or chronic disease was observed, indicating the safety of [^18^F]AlF-PSMA-137.

In view of the quality control results, safety, specificity and affinity to PSMA, the clinical study of [^18^F]AlF-PSMA-137 was performed with the approval of Ethics Committee of Beijing Cancer Hospital. Thirteen patients with newly diagnosed PCa were included and [^18^F]AlF-PSMA-137 PET/CT imaging was conducted.

Similar to the extensively reported Glu-Ureas based probes, [^18^F]AlF-PSMA-137 mainly accumulated in kidneys, lacrimal glands, parotid glands, submandibular glands, liver and bladder, and the uptake were increased in these organs between 1 h p.i. and 2 h p.i. expecting for the bladder. Compared to most PSMA targeted tracers, the bladder showed low accumulation of [^18^F]AlF-PSMA-137 at 2 h p.i., which was similar to ^18^F-PSMA-1007 (33). This was thought to facilitate the detection of intraprostatic lesions, recurrent pelvic lesions, metastatic lymph node lesions and prostate bed lesions. In contrast, ^68^Ga-PSMA-11 is rapidly excreted through the urethra (34), leading to significant accumulation in the bladder, and may somewhat obscure the detection of local recurrences (35).

PET/CT imaging validated its excellent ability to detect the primary and metastatic lesions. Among 13 patients, 81 lesions were detected and over 91% of lesions increased between 1 h p.i. (SUVmean: 10.98 ± 18.12) and 2 h p.i. (SUVmean: 14.25 ± 21.28). Low bone uptake demonstrated the stability in the body as well as availed the detection of the bone metastases. Interestingly, one patient with Gleason score of 4 + 4 showed more than 10 soft tissue metastases. Similar uptake of PSMA targeted tracer in lung metastases have been reported (35, 36). Although [^18^F]AlF-PSMA-137 PET/CT imaging did not change the stage of these prostate cancer patients, it showed low blood uptake and higher tumor-to-organ ratios at delayed time (**Figure 7**), which could improve the imaging contrast. Larger sample sizes are needed to assess the optimal imaging time, and we considered that imaging at 1-2 h is basically appropriate.

The internal radiation dosimetry of 4 PCa patients showed that all organs are tolerated well due to the most organs presented low absorbed doses. The kidneys were the most irradiated organ (0.113 mGy/MBq), which compared to other Glu-Ureas based probes for instance, [^18^F]AlF-PSMA-BCH (0.135 mGy/MBq)[11], ^68^Ga-PSMA-11 (0.262 mGy/MBq reported by Afshar-Oromieh et al. and 0.121 mGy/MBq reported by Pfob et al.) (37, 38), ^18^F-PSMA-1007 (1.70E–01 mGy/MBq) (13) and ^18^F-DCFPyL (0.0945 mGy/MBq) (39). The salivary glands is another dose-limiting organ with an absorbed dose of 5.68E-02 mGy/MBq due to the PSMA expression here (40). The average effective dose of [^18^F]AlF-PSMA-137 was calculated to be 1.19E-02 ± 8.70E-04 mSv/MBq (range: 1.10E-02 - 1.33E-02 mSv/MBq) which means that the 4 PCa patients received an average effective dose of 2.59 ± 0.29 mSv (range: 2.28 - 2.9 mSv).

However, there are several limitations in this study. First, the small sample size was a major limitation of this study, since our initial objective was to share preclinical data and preliminary clinical diagnostic potential of the probe. Second, since this study is the first-in-human imaging, there is no direct comparison with other PSMA tracers, especially fluorine-radiolabeled ones such as ^18^F-SMA-1007 and ^18^F-DCFPyL. Intra-individual and large comparisons will be needed to highlight the potential benefits of each tracer’s characteristics for individual patients (41).

## Conclusion

The [^18^F]AlF^2+^-labeled ODAP-based PSMA probe showed promising PSMA specificity *in vitro* as well as high and targeted uptake *in vivo*. The pilot clinical translational study and radiation dosimetry estimation had confirmed the feasibility and safety of prostate cancer imaging. The detection of new lesions at a delayed time indicated that the [^18^F]AlF-PSMA-137 could be used as a diagnostic PET tracer for monitoring prostate cancer disease.

## Data availability statement

The original contributions presented in the study are included in the article/**Supplementary Material**. Further inquiries can be directed to the corresponding authors.

## Ethics statement

This study was reviewed and approved by Ethics Committee of Peking University Cancer Hospital. The patients/participants provided their written informed consent to participate in this study. The animal study was reviewed and approved by the Peking University Cancer Hospital Animal Care and Use Committee.

## Author contributions

ZY, HZ, PD and XY conceived and designed the experiments. YR and TL performed all of the experiments, data collection and analysis, and wrote the manuscript. CL performed the recruitment of patients, data collection, image analysis and co-wrote the manuscript. XD, QZ, JL, PW and QG were involved in the preparation of radiopharmaceuticals and took part in most of the animal experiments. All authors contributed to the article and approved the submitted version.

## Funding

This study was supported by National Natural Science Foundation of China projects (No. 82102092, No. 21877004, No. 92059101), Beijing Hospitals Authority Dengfeng Project (DFL20191102), the Pilot Project (4th Round) to Reform Public Development of Beijing Municipal Medical Research Institute (2021–1), Capital’s Funds for Health Improvement and Research (2022-1G-1021) and the third foster plan in 2019 “Molecular Imaging Probe Preparation and Characterization of Key Technologies and Equipment” for the development of key technologies and equipment in major science and technology infrastructure in Shenzhen.

## Acknowledgments

We gratefully appreciate all of the chemists, nurses and technicians from the Department of Nuclear Medicine, Peking University Cancer Hospital, for their contributions to tracer administration and PET/CT imaging.

## Conflict of interest

The authors declare that the research was conducted in the absence of any commercial or financial relationships that could be construed as a potential conflict of interest.

## Publisher’s note

All claims expressed in this article are solely those of the authors and do not necessarily represent those of their affiliated organizations, or those of the publisher, the editors and the reviewers. Any product that may be evaluated in this article, or claim that may be made by its manufacturer, is not guaranteed or endorsed by the publisher.
