# Supplementary material for: Preclinical evaluation and first in human study of Al18F radiolabeled ODAP-urea-based PSMA targeting ligand for PET imaging of prostate cancer
Source: Front Oncol. 2022 Oct 20;12:1030187. doi: 10.3389/fonc.2022.1030187 (PMC9633261; doi:10.3389/fonc.2022.1030187)
Supplement: Supplementary file 1 [file DataSheet_1.pdf]

Supplementary Material

1 Results

1.1 Supplementary Figures

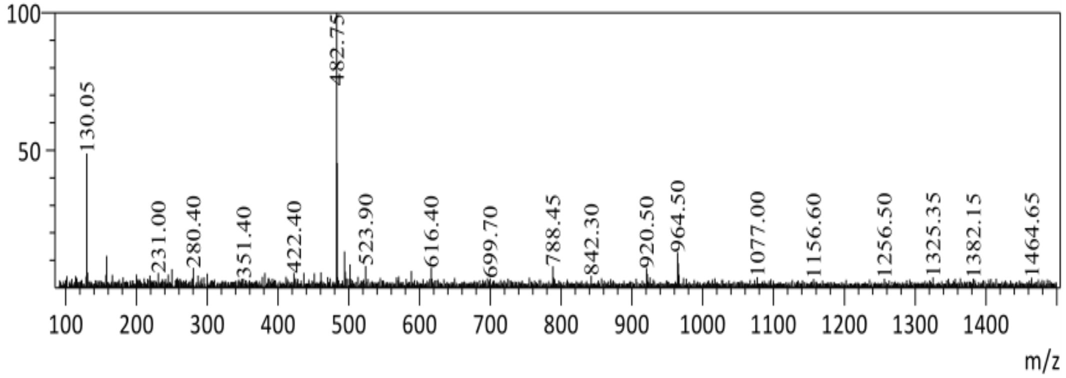

Figure. S 1 ESI-MS chromatogram of NOTA-PSMA-137. The mass spectrum result of NOTA-PSMA-137 was 964.5 ($[M-F]^+$) .

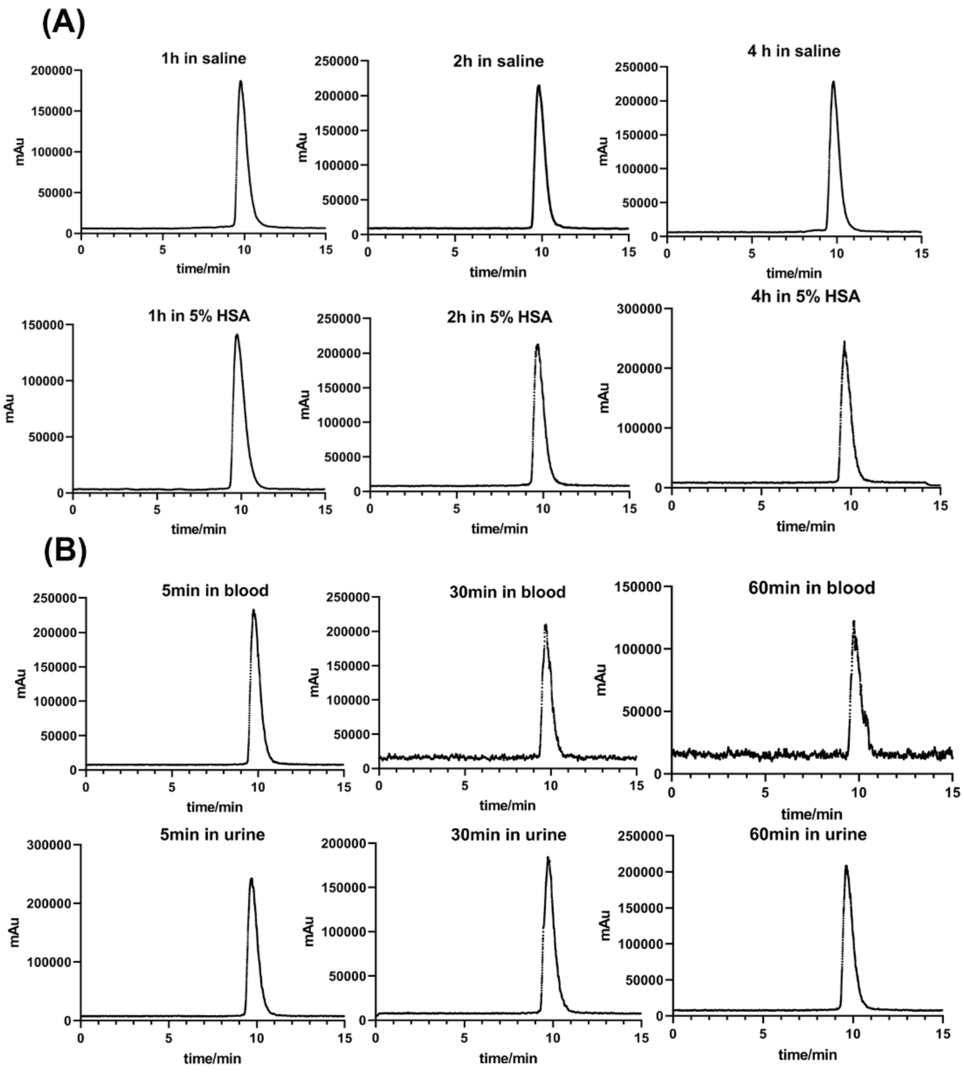

Figure. S 2 **(A)** *Vitro* stability after incubation in saline and 5 % human serum albumin. **(B)** *Vivo* stability in blood and urine of [^{18}F]AlF-PSMA-137.

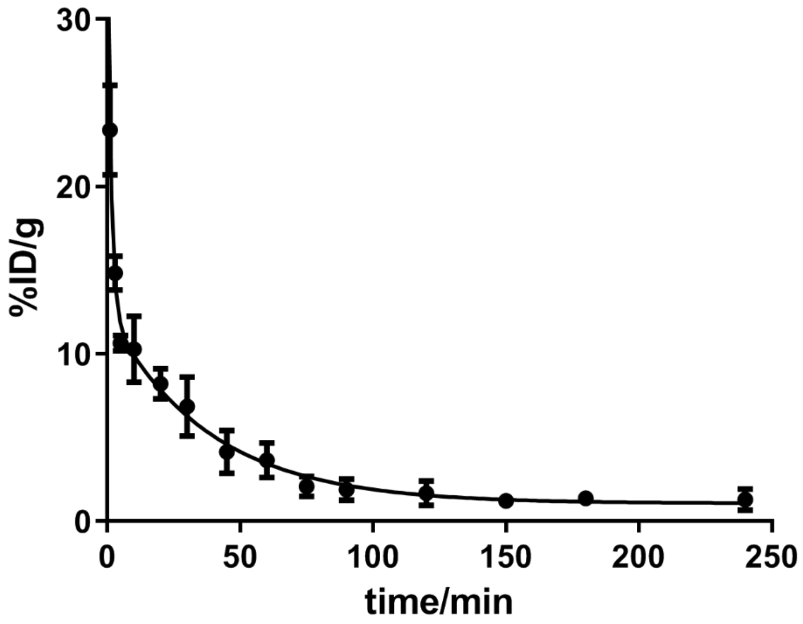

Figure. S 3 The pharmacokinetics of [^{18}F]AlF-PSMA-137 in blood (n = 5). The half-life of distribution phase and elimination phase were 0.961 min and 26.43 min, respectively.

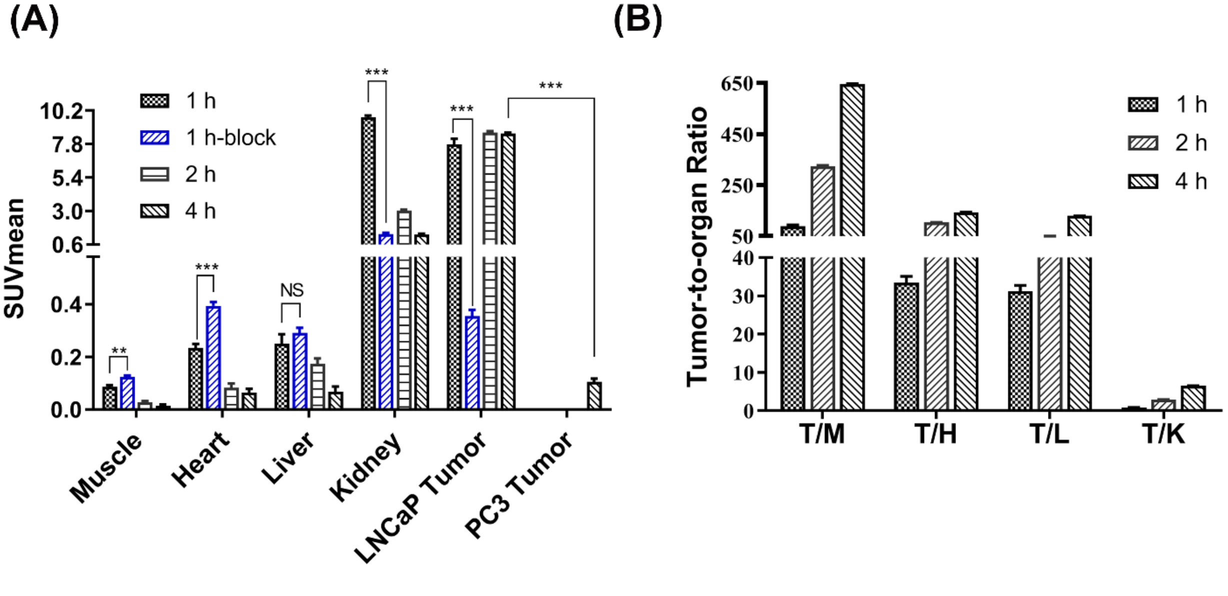

Figure. S 4 **(A)** The SUVmean of kidneys, tumor, heart and liver according to ROIs. **(B)** Ratios of tumor-to-organ according to ROIs. ***: P<0.001.

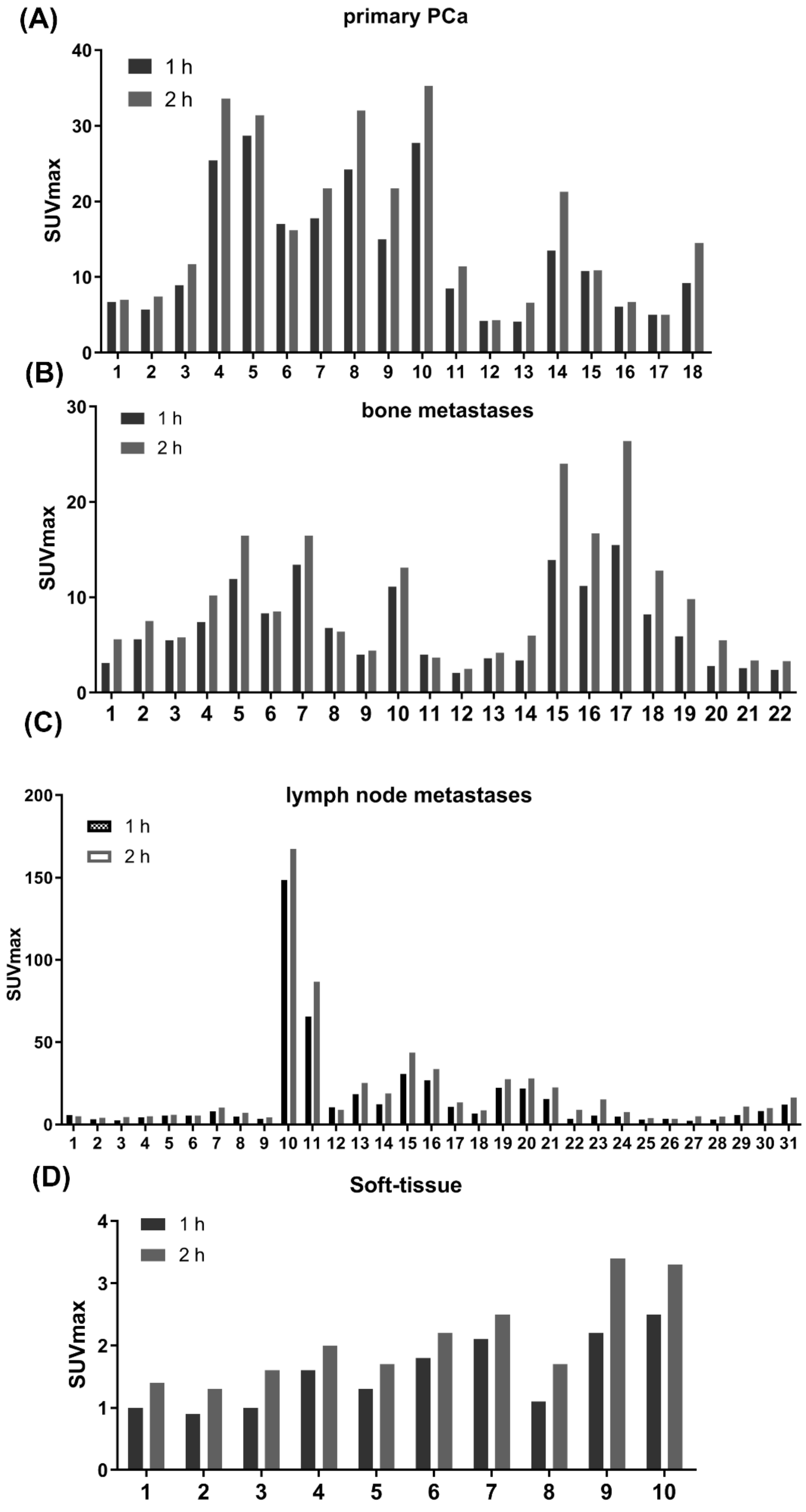
(A)
primary PCa
1 h
2 h
SUVmax
40
30
20
10
0
1 2 3 4 5 6 7 8 9 10 11 12 13 14 15 16 17 18
(B)
bone metastases
1 h
2 h
SUVmax
30
20
10
0
1 2 3 4 5 6 7 8 9 10 11 12 13 14 15 16 17 18 19 20 21 22
(C)
lymph node metastases
1 h
2 h
SUVmax
200
150
100
50
0
1 2 3 4 5 6 7 8 9 10 11 12 13 14 15 16 17 18 19 20 21 22 23 24 25 26 27 28 29 30 31
(D)
Soft-tissue
1 h
2 h
SUVmax
4
3
2
1
0
1 2 3 4 5 6 7 8 9 10

Figure. S 5 The SUVmax in lesions including 18 primary PCa **(A)**, 22 bone metastases **(B)**, 31 lymph node metastases **(C)** and 10 soft-tissue **(D)** at 1 h p.i. and 2 h p.i..

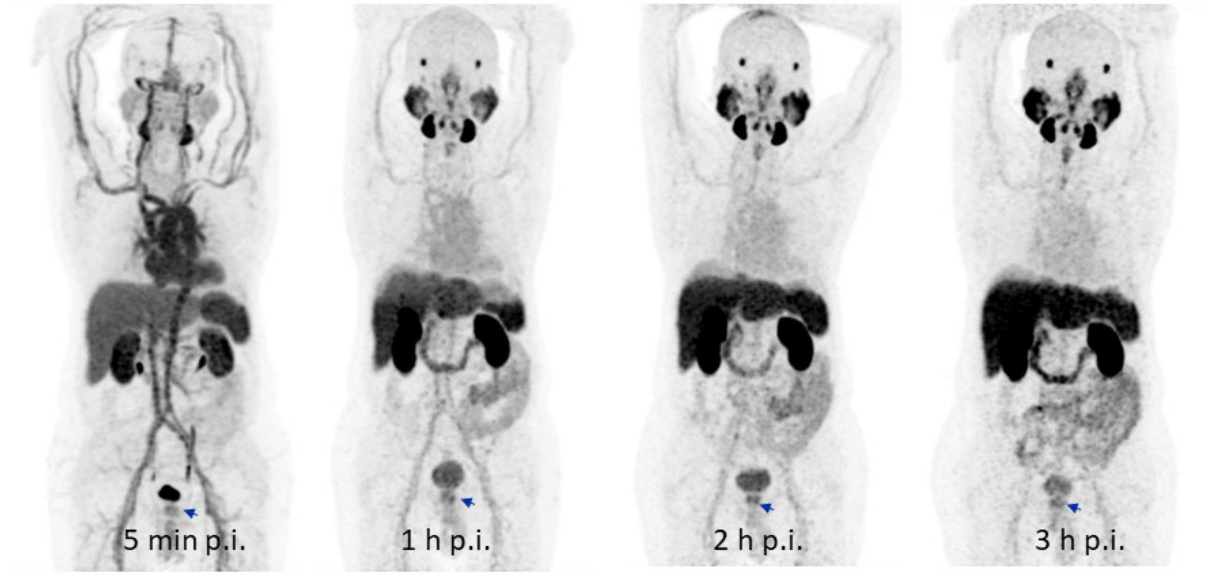

Figure. S 6 Maximum-intensity projections in a 72 years man of No.10 with PSA of 21.49 ng/mL and Gleason score of 4+5=9 at 5 min, 1 h p.i., 2 h p.i. and 3 h p.i.. The blue arrows indicated primary PCa (SUVmax: 5.9, 6.1, 6.7 and 6.3 at 5 min, 1 h p.i., 2 h p.i. and 3 h p.i.).

1.2 Supplementary Tables

Table S 1 Uptake value of [^{18}F]AlF-PSMA-137 in mice bearing LNCaP tumors at 1 h p.i.. (% ID/g, mean ± SD, n = 4)

Organ	no blockade	blockade	P value
Blood	1.39 ± 0.31	1.26 ± 1.09	0.8570
Heart	0.76 ± 0.14	0.54 ± 0.29	0.2838
Liver	0.72 ± 0.08	0.91 ± 0.82	0.7016
Spleen	2.82 ± 0.85	0.67 ± 0.37	0.0070
Lung	2.07 ± 0.20	1.52 ± 1.13	0.4340
Kidney	75.19 ±20.04	2.39 ± 1.92	0.0017
Stomach	0.24 ± 0.07	0.47 ± 0.39	0.3529

Small intestine	1.30 ± 0.54	1.45 ± 0.69	0.7850
Large intestine	1.06 ± 0.27	0.88 ± 0.44	0.5658
Muscle	0.95 ± 0.11	0.56 ± 0.26	0.0562
Brain	0.11 ± 0.01	0.07 ± 0.03	0.0747
Tumor	24.29 ± 3.06	2.09 ± 0.81	0.0006
Tumor/muscle	23.31± 2.15	-	-
Tumor/kidney	0.37 ± 0.07	-	-

Table S 2 Biodistribution of [^{18}F]AlF-PSMA-137 in PCa patients (mean ± SD, n = 13)

organ	SUVmax		SUVmean	
	1 h	2 h	1 h	2 h
brain	0.36 ± 0.22	0.34 ± 0.17	0.07 ± 0.04	0.07 ± 0.03
lacr. gland	8.45 ± 2.65	10.96 ± 3.10	4.31 ± 0.91	4.97 ± 0.93
par. gland	13.5 ± 3.27	17.41 ± 3.37	8.83 ± 1.74	11.20 ± 2.60
sub. gland	15.17 ± 3.53	18.88 ± 3.30	9.31 ± 2.13	10.95 ± 2.82
thyroid gland	1.78 ± 0.41	1.37 ± 0.32	1.28 ± 0.26	1.02 ± 0.21
heart	3.77 ± 0.39	3.18 ±0.67	2.88 ± 0.43	2.26 ± 0.27
lung	1.03 ± 0.18	0.98 ± 0.2	0.65 ± 0.07	0.61 ± 0.12

liver	12.78 ± 4.03	14.52 ± 4.01	8.59 ± 1.21	9.58 ± 1.56
stomach	2.26 ± 0.58	2.18 ± 0.73	1.48 ± 0.52	1.27 ± 0.60
spleen	6.63 ± 2.18	6.75 ± 2.16	5.37 ± 1.87	5.19 ± 1.93
small int.	1.84 ± 0.66	1.67 ± 0.68	1.27 ± 0.45	1.11 ± 0.40
pancreas	2.69 ± 0.30	2.19 ± 0.40	1.93 ± 0.27	1.57 ± 0.27
kidney	20.55 ± 5.71	26.69 ± 7.20	10.67 ± 2.17	13.52 ± 2.93
rectum	4.43 ± 4.10	2.41 ± 0.58	2.35 ± 1.38	1.59 ± 0.48
bladder	12.62 ± 8.34	6.70 ± 2.16	8.34 ± 5.50	5.02 ± 2.06
muscle	0.80 ± 0.15	0.72 ± 0.20	0.55 ± 0.06	0.48 ± 0.12
bone	0.87 ± 0.41	0.79 ± 0.29	0.61 ± 0.20	0.46 ± 0.15
primary PCa	13.25± 8.17	16.59 ± 10.32	-	-
LN metastases	15.57 ± 27.26	20.06 ± 31.49	-	-
bone metastases	6.94 ± 4.09	9.67 ± 6.58	-	-
soft-tissue	1.55 ± 0.55	2.11 ± 0.71		

lacr. = lacrimal; par. = parotid; sub. = submandibular; int. = intestine.

Table S 3 Dosimetry estimation of [^{18}F]AlF-PSMA-137 in 4 PCa patients.

Target Organ	Absorbed Dose (mGy/MBq)			
	No.10	No.11	No.12	No.13
Adrenals	3.10E-02	2.45E-02	2.17E-02	2.16E-02
Brain	2.90E-03	2.46E-03	2.19E-03	2.52E-03
Esophagus	1.06E-02	8.76E-03	8.91E-03	9.26E-03
Eyes	4.58E-03	3.38E-03	3.40E-03	4.66E-03
Gallbladder Wall	2.77E-02	4.29E-02	4.30E-02	3.19E-02
Left colon	1.06E-02	8.08E-03	7.39E-03	9.11E-03
Small Intestine	9.21E-03	6.86E-03	6.50E-03	8.43E-03
Stomach Wall	1.89E-02	1.48E-02	1.06E-02	1.20E-02
Right colon	1.10E-02	8.77E-03	8.46E-03	9.70E-03
Rectum	7.19E-03	5.16E-03	5.17E-03	7.60E-03
Heart Wall	2.14E-02	1.88E-02	2.38E-02	1.99E-02
Kidneys	1.51E-01	1.13E-01	9.55E-02	9.06E-02
Liver	5.21E-02	4.36E-02	4.35E-02	3.66E-02
Lungs	1.24E-02	1.32E-02	1.41E-02	1.13E-02

Pancreas	1.22E-02	1.03E-02	9.34E-03	9.86E-03
Prostate	7.66E-03	5.56E-03	5.58E-03	8.21E-03
Salivary Glands	4.49E-02	6.47E-02	6.71E-02	5.03E-02
Red Marrow	7.21E-03	5.65E-03	5.81E-03	6.77E-03
Osteogenic Cells	1.07E-02	8.80E-03	1.20E-02	1.14E-02
Spleen	5.44E-02	5.91E-02	3.32E-02	4.65E-02
Testes	5.24E-03	3.65E-03	3.58E-03	5.51E-03
Thymus	8.54E-03	6.97E-03	7.62E-03	8.12E-03
Thyroid	1.57E-02	1.61E-02	1.14E-02	1.65E-02
Urinary Bladder Wall	1.56E-02	1.31E-02	1.75E-02	2.84E-02
Total Body	7.98E-03	6.13E-03	6.10E-03	7.42E-03
Effective Dose (mSv / MBq)	1.33E-02	1.18E-02	1.10E-02	1.14E-02
